# Validation of the Portuguese version of Amsterdam Preoperative Anxiety and Information Scale (APAIS)

**DOI:** 10.1186/s12955-021-01736-6

**Published:** 2021-03-19

**Authors:** Sandra Maurício, Isabel Rebêlo, Catarina Madeira, Filipa Resende, Susana Esteves

**Affiliations:** 1 Serviço de Anestesiologia, Instituto Português de Oncologia de Lisboa, Lisbon, Portugal; 2 Unidade de Investigação Clínica, Instituto Português de Oncologia de Lisboa, Lisbon, Portugal

**Keywords:** Anxiety, Cancer, Surgery, Anesthesia, Questionnaire, Validation study, Perioperative care

## Abstract

**Background:**

Preoperative anxiety is common among the oncological surgical population. Due to its psychological and physiological detrimental effects, identifying and addressing it is of uttermost importance to improve anesthetic management and patient’s outcomes. The aim of this study is to validate the Portuguese version of Amsterdam Preoperative Anxiety and Information Scale (APAIS) in the oncological population.

**Methods:**

Following forward and backward translation of the original APAIS scale, further adaptation was obtained through cognitive interviewing. The resulting instrument was tested on the day before surgery on a sample of adult cancer surgical patients from a Portuguese oncology centre. Psychometric evaluation was derived from inter-item correlation, confirmatory factor analysis, Cronbach’s alpha, correlation with comparative scales, receiver operating characteristic curve and Youden index.

**Results:**

109 patients (58 males, 51 females) were included. A three-dimensional model—anxiety about anesthesia, anxiety about surgery and desire for information, showed the best fit to the data. The questionnaire revealed high internal consistency (Cronbach alpha 0.81) and good inter-item correlation. Also, Portuguese APAIS correlated well with the gold standard anxiety scale. Therefore, the psychometric properties of this scale version make it a valid and reliable instrument. The optimal cutoff to maximize both sensitivity and specificity was 12 for the APAIS global anxiety score.

**Conclusions:**

Portuguese APAIS version is an accurate tool to identify preoperative anxiety among cancer patients and might impact its management, from premedication choice to provision of information and reassurance about either anesthesia or surgery.

## Background

Preoperative anxiety has been recognized as a significant issue among surgical patients [1, 2]. Excessive anxiety leads to psychological and physical adverse stress reactions. It causes a greater consumption of anesthetic [3, 4] and analgesic drugs [5], intra- and post-operatively. Moreover, it is a negative predictor of surgical outcomes [6] and has a deleterious impact on the patient’s health care experience [7].

Preoperative anxiety is a multifactorial disorder. Causes may include fear of anesthesia, concern about surgical outcomes, anticipation of post-operative pain and hospital environment hostility [8]. It is also influenced by socio-demographic and psychosocial factors, which may be of great significance and challenging to evaluate [9, 10].

Regarding the oncological population, 18% of the patients report depression symptoms and 24% anxiety symptoms [11]. In the preoperative setting, these patients have additional anxiety causes, including the life-threatening nature of their disease, the surgical impact on their body image and the effects of other non-surgical treatments [12]. Psychological pre-rehabilitation has been suggested to have a favorable outcome in improving postoperative functional capacity and resuming normal daily activities [13]. However, albeit the relevance of psychological factors in postsurgical outcomes, these variables are not systematically evaluated. As a result, despite being well known among clinicians, anxiety is not being sufficiently treated.

Several instruments can be used to evaluate patients’ anxiety. The *State-Trait Anxiety Inventory* (STAI) and the *Hospital Anxiety and Depression Scale* (HADS) [14] have been widely used in the hospital setting. They both have already been translated and validated to the Portuguese language. However, they are long and time-consuming, have not been designed for the pre-operative setting and do not assess the need for information.

The *Amsterdam Preoperative Anxiety and Information Scale* (APAIS) was created in order to obtain a rapid and easy evaluation of the patient’s anxiety and need for information about the surgery and anesthesia [15]. The APAIS questionnaire has been showing remarkable results in the preoperative evaluation of subjects undergoing a variety of procedures, from minor to major surgery, as it allows the identification of patients who would benefit from therapeutic intervention and/or further information [16]. The APAIS has been translated into several languages [17–22], but it has not been validated for the Portuguese population neither specifically for cancer patients.

The aim of this study is to translate the APAIS into Portuguese and validate this version for the oncologic population.

## Methods

### Design, setting and ethics

Following the institution’s Ethics Committee approval, this cross-sectional psychometric study was carried out at Instituto Português de Oncologia de Lisboa Francisco Gentil, one of the main oncology centers in Portugal. Participant selection was done using a quota sampling procedure to obtain equal proportion of males and females. Patients were recruited between July and December 2018, and written consent was obtained from all of them.

### The scale

The APAIS is a simple questionnaire consisting of six items, four regarding the anxiety about the surgical procedure and the fear of anesthesia, and two regarding the need for information (Table 1). The answers are recorded in a 5-point *Likert* scale, thus the anxiety scale ranges from 4 to 20 points and the need for information scale ranges from 2 to 10 points. The original cutoffs from the Dutch version and its English translation are 11 for the clinical context, and 13 for investigation purposes due to its higher specificity [15].

**Table 1:** Items of the *Amsterdam Preoperative Anxiety and Information Scale*

English (Moerman et al. [15])	Portuguese (before cognitive interviews)	Portuguese (final version)
1. I am worried about the anesthesia	1. Estou preocupado com a anestesia	1. Estou preocupado com a anestesia
2. The anesthesia is on my mind continuously	2. Estou continuamente a pensar na anestesia	2. Estou permanentemente a pensar na anestesia
3. I would like to know as much as possible about the anesthesia	3. Gostaria de saber o mais possível sobre a anestesia	3. Gostaria de saber mais sobre a anestesia
4. I am worried about the procedure	4. Estou preocupado com a cirurgia	4. Estou preocupado com a cirurgia
5. The procedure is on my mind continuously	5. Estou continuamente a pensar na cirurgia	5. Estou permanentemente a pensar na cirurgia
6. I would like to know as much as possible about the procedure	6. Gostaria de saber o mais possível sobre a cirurgia	6. Gostaria de saber mais sobre a cirurgia

### Translation and adaptation

First, authorization to translate the APAIS was required from the authors of the original scale. According to the WHO guidelines for translation and adaptation of instruments [23], the process included a forward translation by two anesthesia trainees, an expert review by two bilingual senior anesthetists, a backward translation by an independent translator and cognitive interviewing with ten surgical adult inpatients by a senior clinical psychologist, in order to obtain an equivalent scale in Portuguese.

### Sampling process, study subjects and data collection

The size of the validation sample was based on a ratio of 20 cases per item, which holds a number that is similar to those seen in other APAIS validation studies [17–22]. The sample included patients aged above 18 years old, able to read and understand the Portuguese language, undergoing elective cancer surgery, either general, gynaecological or urological procedures. Exclusion criteria were: psychiatric disorder requiring antipsychotic drugs, cognitive impairment, difficulty understanding the study, non-cancer or emergent surgery. The day before surgery, and after the pre-anesthetic visit, patients filled in the questionnaires.

### Patients’ characteristics and comparative scales

Patients’ sociodemographic and clinical variables potentially associated with anxiety and desire for information were collected. These included age, gender, ASA physical status, education, work situation and marital status, previous anesthesia and/or surgery, regular use of anxiolytics, type of surgery and waiting time. The instruments used were the APAIS (anxiety scale—4 items, score 4–20; need for information scale—2 items, score 2–10), STAI-Y1 (subscale for anxiety-state—20 items, score 20–80, [24]) and HADS (subscale A for anxiety and subscale D for depression—7 items each, score 0–21, [25]) for post hoc comparisons.

### Statistical analysis and psychometric evaluation

We performed a descriptive analysis of the sociodemographic and clinical variables and of the APAIS, STAI-Y1, HADS-A and HADS-D scores using mean, standard deviation (SD), and absolute and relative frequencies.

Evaluation of APAIS validity was conducted as follows. A confirmatory factor analysis (CFA) has been performed to evaluate whether the APAIS Portuguese version, once applied to oncology patients, would maintain the same factor structure reported in previous validation studies. We evaluated the one-factor model as described in the Spanish APAIS validation [22], the two-factor model as in the original APAIS [15] and the three-factor model as in the French APAIS validation [19]. Kaiser–Meyer–Olkin measure of sampling adequacy (0.77) and Bartlett's test of sphericity (*p* < 0.001) confirmed data suitability for factor analysis but Mardia’s test rejected multivariate normality. Therefore, we used a maximum likelihood estimation with robust standard errors and a Satorra–Bentler scaled test statistic suitable for non-normal data. We report the fit indices derived from this robust approach chi-square test of exact fit (*p* > 0.05 indicates good fit), Tucker Lewis Index (good fit if TLI ≥ 0.95), comparative fit index (good fit if CFI ≥ 0.95) and the root mean square error of approximation (good fit if RMSEA < 0.08) and its 90% confidence interval (90% CI). The Scaled Chi Square Difference Test was used for model comparison (likelihood ratio test with robust estimation).

Internal consistency of the APAIS scale was assessed by Cronbach's alpha coefficient (reliability acceptable if ≥ 0.70). Nonparametric Spearman correlations were used for determining the associations and correlations between the scales, with the correlation between STAI-Y1 and APAIS global anxiety score (resulting from the sum of scores from items 1, 2, 4 and 5) being used to evaluate concurrent validity. Finally, we attempted to identify the utility of the APAIS in Portuguese cancer patients. The sensitivity, specificity, and positive and negative predictive values of the APAIS global anxiety score were assessed for different cutoff points by using a STAI-Y1 score ≥ 40 as the reference point to detect clinically significant anxiety [26]. Accuracy was estimated by receiver operating characteristic (ROC) curve and the corresponding area under the curve (AUC). The best cutoff of APAIS was determined by analysis of accuracy at every APAIS score and by Youden index.

We considered the significance level of 5% unless otherwise specified. The statistical analysis was performed using R [27] and the packages “lavaan” [28], “psych” [29], “pROC” [30] and “epiR” [31].

## Results

### Process of translation and adaptation

Following translation into Portuguese by a Portuguese anesthesiologist knowledgeable of the English language, the scale was reviewed by a different bilingual health professional. Then, it was independently translated back into English with no mismatches. Further feedback was obtained from pretesting using cognitive assessment. Ten surgical patients were interviewed by a clinical psychologist with experience in psycho-oncology and instrument development. Cognitive interviewing included participant rephrasing of the original sentences, inquiries about words and expressions that might sound confusing, offensive or upsetting. According to the participants, direct translation on items 2, 3, 5 and 6 did not describe their experience and context properly. Participant suggestions were then considered: “continuamente” (continuously) was replaced by “permanentemente” (permanently) in items 2 and 5, “o mais possível” (as much as possible) was replaced by “mais” (more) in items 3 and 6. The six items of the original English version and their Portuguese equivalents are shown in Table 1.

### Characteristics of study subjects

123 subjects were recruited, although fourteen of them have withdrawn from the study due to difficulties in interpretation and filling the three distinctive written questionnaires. Low literacy levels, as explained below, might have discouraged these patients from participating. Hence the response rate among the included subjects was 100% and there was no missing data, which indicates good acceptability of all questions. Characterisation of the respondent group is presented in Table 2. Men represented 53% of the sample, median age was 62 years and 95% had an ASA status score of II or higher. 43% of the patients had only attained primary school education, less than half were currently employed and the majority were married. Additionally, 83% of the study participants had been anesthetized before and 71% had undergone surgery before. Regarding waiting times, 74% of the patients had waited over a month for cancer surgery. Anxiolytics were used regularly by 11% of respondents, roughly in accordance with general population data [32].

**Table 2:** Characteristics of the respondents

Characteristics	Male	Female	Total
Respondents, *no. (%)*	58 (53)	51 (47)	109 (100)
Age in years, *median (Q1–Q3)*	64 (53–72)	57 (50–66)	62 (50–68)
*Physical status*			
ASA I, *no. (%)*	2 (3)	3 (6)	5 (5)
ASA II, *no. (%)*	32 (55)	35 (69)	67 (61)
ASA III, *no. (%)*	24 (41)	13 (25)	37 (34)
*Education*			
None, *no. (%)*	1 (2)	0 (0)	1 (1)
Basic, *no. (%)*	26 (45)	21 (41)	47 (43)
Secondary, *no. (%)*	21 (36)	14 (27)	35 (32)
Tertiary, *no. (%)*	10 (17)	16 (31)	26 (24)
*Work situation*			
Employed, *no. (%)*	24 (41)	25 (49)	49 (45)
Unemployed, *no. (%)*	4 (7)	9 (18)	13 (12)
Retired, *no. (%)*	30 (52)	17 (33)	47 (43)
*Marital status*			
Single, *no. (%)*	10 (17)	6 (12)	16 (15)
Married, *no. (%)*	41 (71)	34 (67)	75 (69)
Divorced, *no. (%)*	4 (7)	5 (10)	9 (8)
Widowed, *no. (%)*	3 (5)	6 (12)	9 (8)
Previous anesthesia, *no. (%)*	47 (81)	44 (86)	91 (83)
Previous surgery, *no. (%)*	38 (66)	39 (76)	77 (71)
Regular use of anxiolytics, *no. (%)*	4 (7)	8 (16)	12 (11)
*Type of surgery*			
General, *no. (%)*	40 (69)	29 (57)	69 (63)
Gynecologic, *no. (%)*	–	19 (37)	19 (17)
Urologic, *no. (%)*	18 (31)	3 (6)	21 (19)
*Waiting time for surgery*			
< 1 month, *no. (%)*	10 (17)	18 (35)	28 (26)
1–3 months, *no. (%)*	26 (45)	29 (57)	55 (50)
3–6 months, *no. (%)*	12 (21)	3 (6)	15 (14)
> 6 months, *no. (%)*	10 (17)	1 (2)	11 (10)

### Scales scoring and internal consistency of the APAIS

As shown in Table 3, the mean scores were as follows: APAIS anxiety 12.82 ± 4.68, APAIS desire for information 7.33 ± 2.29, STAI-Y1 42.10 ± 10.59. Of the 109 patients, 61% would be classified as anxious using STAI-Y1 (score ≥ 40), and 34% using HADS-A. 18% were found to be at least mildly depressed using HADS-D [25]. Overall APAIS items showed good inter-item correlations, the weakest correlation was between item 6 and items 1 and 2 (Table 4).

**Table 3:** Scales scoring (n = 109)

Scales and items	Scores, *mean (SD)*		
*APAIS*			
Item 1	3.15 (1.55)	Anxiety about anesthesia 5.49 (2.82)	Anxiety 12.82 (4.68)
Item 2	2.34 (1.52)		
Item 4	3.99 (1.37)	Anxiety about surgery 7.33 (2.53)	
Item 5	3.34 (1.45)		
Item 3	3.48 (1.42)		Desire for information 7.33 (2.29)
Item 6	3.85 (1.36)		
*STAI*			
Anxiety-state (Y1)	42.10 (10.59)		
*HADS*			
Anxiety (A)	6. 37 (3.99)		
Depression (D)	4.07 (3.67)

**Table 4:** Inter-item correlation matrix

Items	1	2	3	4	5	6
1. I am worried about the anesthesia	1.00					
2. The anesthesia is on my mind continuously	0.70	1.00				
3. I would like to know as much as possible about the anesthesia	0.45	0.43	1.00			
4. I am worried about the procedure	0.48	0.42	0.31	1.00		
5. The procedure is on my mind continuously	0.44	0.51	0.38	0.62	1.00	
6. I would like to know as much as possible about the procedure	0.19	0.12	0.31	0.34	0.40	1.00

### Tests of dimensionality

Confirmatory factor analysis was run, evaluating three a priori hypotheses: one-factor model (as in the Spanish APAIS validation), two-factor model (as in the original APAIS) and three-factor model (as is the French APAIS validation). Overall, the model with the best fit to the data was a three-dimensional model: anxiety about anesthesia, anxiety about surgery, desire for information. Table 5 reports fit statistics for each model.

**Table 5:** Confirmatory factor analysis adjustment parameters

Models and items	1-factor model 1 + 2 + 3 + 4 + 5 + 6	2-factor model Anxiety 1 + 2 + 4 + 5 Desire for information 3 + 6	3-factor model Anxiety about anesthesia 1 + 2 Anxiety about surgery 4 + 5 Desire for information 3 + 6
Chi-square p	< 0.001	< 0.001	0.066
TLI	0.796	0.776	0.926
CFI	0.878	0.880	0.970
RMSEA (90% CI)	0.156 (0.110–0.206)	0.164 (0.115–0.217)	0.095 (0.017–0.161)
Model comparison	1-factor versus 2-factor model: *p* = 0.1817 1-factor versus 3-factor model: *p* < 0.001 2-factor versus 3-factor model: *p* < 0.001		

*CFI* comparative fit index, *RMSEA* root mean square error of approximation, *TLI* tucker Lewis index

### Tests of reliability

Regarding the internal consistency of scale items, all items showed item-to-total correlations > 0.5, and Cronbach’s alpha was 0.81, 95% CI 0.71–0.91 (Table 6).

**Table 6:** Item-to-total correlations

Items	Item-to-total correlation (corrected for item overlap)	Guttman’s Lambda 6 (squared multiple correlation)	Cronbach’s alpha (if item removed)
1. I am worried about the anesthesia	0.713	0.754	0.769
2. The anesthesia is on my mind continuously	0.704	0.752	0.771
3. I would like to know as much as possible about the anesthesia	0.582	0.794	0.790
4. I am worried about the procedure	0.661	0.771	0.779
5. The procedure is on my mind continuously	0.737	0.755	0.762
6. I would like to know as much as possible about the procedure	0.481	0.805	0.813

### Tests of convergent validity

**Fig. 1:**
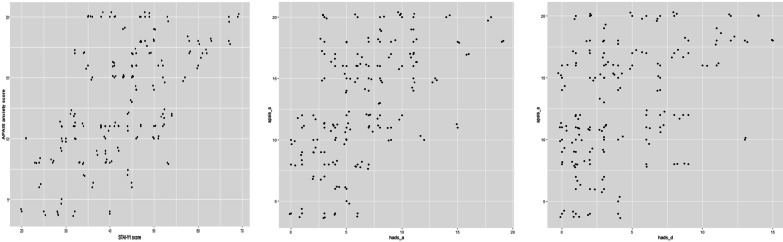
Correlation between APAIS global anxiety score and (**a**) STAI-Y1, (**b**) HADS-A, (**c**) HADS-D

In order to assess the extent to which the Portuguese APAIS version measures anxiety, the APAIS global anxiety score resulting from the sum of scores from items 1, 2, 4 and 5 was compared with STAI-Y1, HADS-A and HADS-D scores (Fig. 1). APAIS score correlates slightly better with the gold standard STAI-Y1 (Spearman’s rho 0.580, *p* < 0.001) and HADS-A as well (Spearman rho 0.539, *p* < 0.001), than with HADS-D results (Spearman’s rho 0.455, *p* < 0.001), which screens for probable depression in the hospital setting.

### Tests of criterion validity

**Fig. 2:**
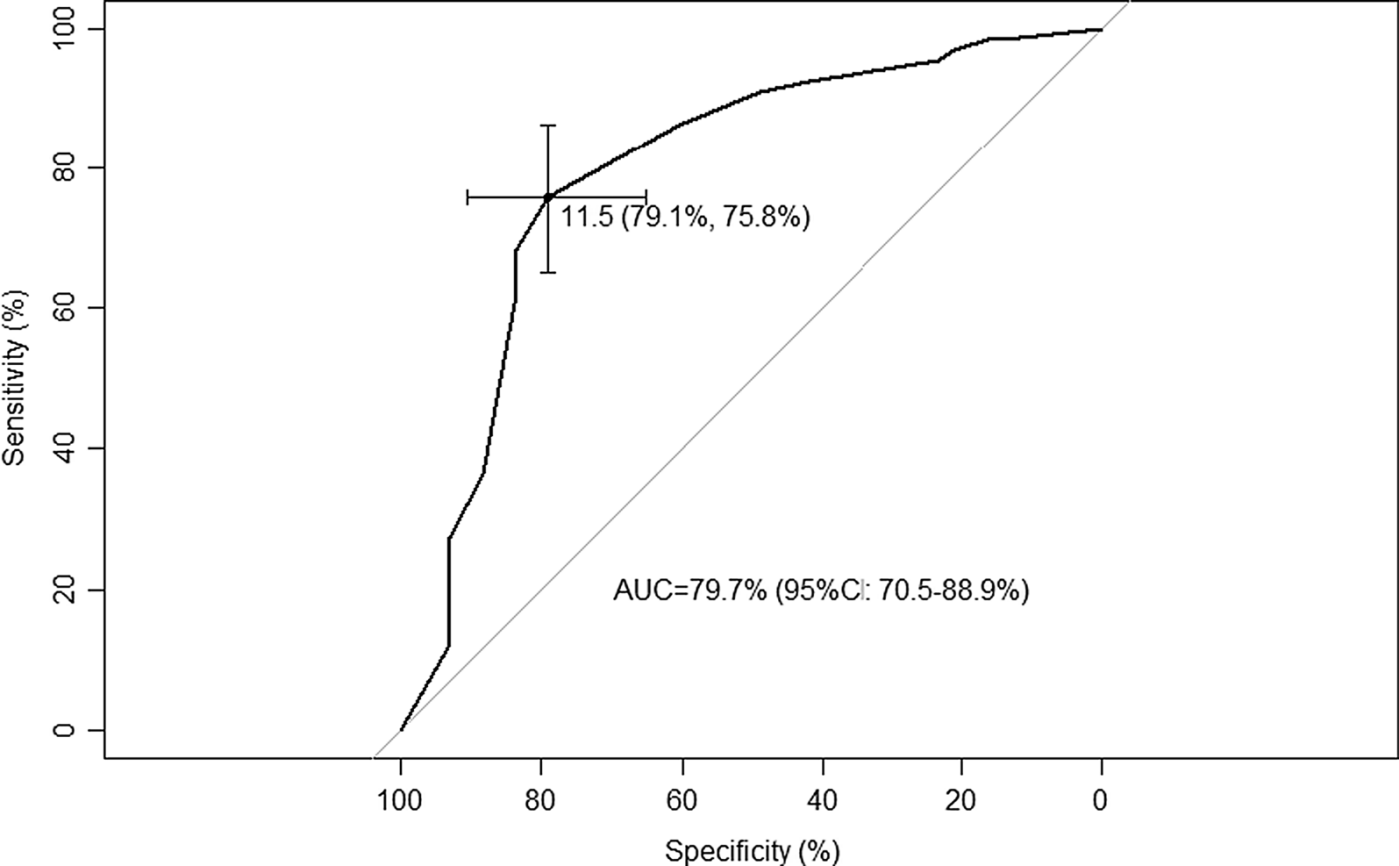
ROC curve for AUC and cutoff point determination

Criteria validity was tested by means of a ROC curve from APAIS global anxiety score and STAI-Y1 scores, yielding an area under the curve for anxiety as assessed by APAIS of 79.7% (95% CI 70.5–88.9%, Fig. 2). The optimal cutoff to maximize sensitivity and specificity was 11.5 (Fig. 2) and several integer cutoff points were tested (Table 7). Overall, a value of 12 is the best cutoff value for the Portuguese version of APAIS until new representative data are available.

**Table 7:** Sensitivity, specificity, predictive values and likelihood ratios for different cutoff values

Metric *(95% CI)*	Cutoff 10	Cutoff 11	Cutoff 12	Cutoff 13
Sensitivity	0.91 (0.81, 0.97)	0.86 (0.76, 0.94)	0.76 (0.64, 0.85)	0.68 (0.56, 0.79)
Specificity	0.49 (0.33, 0.65)	0.60 (0.44, 0.75)	0.79 (0.64, 0.90)	0.84 (0.69, 0.93)
Positive predictive value	0.73 (0.62, 0.82)	0.77 (0.66, 0.86)	0.85 (0.73, 0.93)	0.87 (0.74, 0.94)
Negative predictive value	0.78 (0.58, 0.91)	0.74 (0.57, 0.88)	0.68 (0.53, 0.80)	0.63 (0.49, 0.76)
Positive likelihood ratio	1.78 (1.31, 2.40)	2.18 (1.49, 3.20)	3.62 (1.99, 6.57)	4.19 (2.08, 8.41)
Negative likelihood ratio	0.19 (0.08, 0.42)	0.23 (0.12, 0.43)	0.31 (0.19, 0.48)	0.38 (0.26, 0.55)

## Discussion

The aim of this study is to translate and validate the APAIS for the Portuguese oncologic population. The use of a quick and easy-to-administer instrument to evaluate anxiety is of foremost importance in the perioperative setting as anxiety leads to a more challenging anesthetic management and a worse patient experience. Our study revealed an anxiety prevalence of 61%, higher than other published oncological reports.

The education level of our sample was in line with the average elder Portuguese population (52% of the people aged 65 and over only attained primary school education, PORDATA 2018). Thus, in order to obtain a better comprehension, warranting an appropriate phrasing was crucial. Following WHO guidelines, after cross-validation of the direct translation, the final questionnaire resulted from additional cognitive interviewing. Nevertheless, due to the very low literacy among the elderly, 11% of the participants withdrew from the study.

Contrary to previous validations, all questionnaires were applied after the patient’s admission on the day before surgery, instead of immediately before the surgery. We believe this provided a less stressful environment for the patients.

Results showed high-quality psychometric properties. Scale reliability revealed a Cronbach’s alpha of 0.81, consistent with the one obtained on the original scale and further validations. In contrast, confirmatory factor analysis suggested a three-dimensional model (anxiety about anesthesia, anxiety about surgery, desire for information) as the best fit, differently from the original scale. This model has already been described for the French scale validation [19]. Differences might be explained by cultural and educational reasons, as well as the life-changing nature of the oncological disease and its surgical treatment. Thus inter-item correlation was moderate to high on both anxiety dimensions. A weaker correlation, however, was observed in the desire for information dimension. A paternalistic doctor-patient relationship, based on trustworthiness, may be a reason for the mismatch between reported anxiety and need for information.

Spearman’s correlation confirmed the capacity for the Portuguese version of the APAIS to explore anxiety states. APAIS correlated slightly better with the STAI-Y1 and HADS-A scores (Spearman’s rho 0.580 and 0.539) than with HADS-D (Spearman’s rho 0.455). Albeit the presence of weak correlations among anxiety scales, HADS-D scores were not as disparate as anticipated. As shown in the HADS-D final scores, 18% of the studied population also suffered from depression. Although clinically different, anxiety and depression are both frequent comorbidities in oncological patients, and sometimes coexist, which may justify our observations. Indeed, results from correlation testing between APAIS and HADS-D suggested that preoperative anxiety is usually present in cancer patients suffering from depression (Fig.1b), an association that should be tested in future works.

For this specific population, two cutoffs determined by the ROC curves could be used. For a higher sensitivity (0.86) a cutoff of 11 would be recommended, similar to the original version. However, taking all the results into account, a cutoff of 12 is suggested in order to gain specificity (specificity 0.79).

That said, this study presents some limitations. First, the studied population is restricted to a single hospital and it only includes cancer surgery patients. As previously mentioned, cancer patients are known to have higher baseline anxiety and depression. Furthermore, many of these patients undergo multiple diagnostic and therapeutic procedures during the course of the disease, somehow modulating their perioperative-related anxiety. Secondly, several patients were not included due to low literacy, meaning that further works should be developed in order to evaluate the applicability of a verbal assessment in this group. One factor contributing to the difficulty of answering the questionnaires was their length, particularly the ones used for comparative measures, not APAIS itself. Also, convenience sampling was applied in this study. Last, and unintentionally, no ASA IV or V patients were sampled from the population.

## Conclusion

This study established the Portuguese version of the APAIS as a valid and reliable instrument for the evaluation of preoperative anxiety in cancer patients.

Routine preoperative anesthetic evaluation should encompass identification of not only anxious patients but also those that need further information. In this setting, and with increasing awareness of the impact of different perioperative factors—as anxiety—on overall patient’s experience, a prompt and easy evaluation tool has become of pivotal importance. Recognizing anxiety and need for information is the first step toward clinical intervention, either prescription of anxiolytic premedication or further information and reassurance.

Additionally, as data on interventions to decrease preoperative anxiety are scarce and benzodiazepines continue to be widely used despite its side effects, the application of the APAIS might also be appropriate for evaluating the role of current pharmacological and psychological interventions. Its ability to objectively identify states of anxiety makes it suitable not only for the clinical setting, but also as a tool for further works in this field.

Finally, if meant to be used more widely, the Portuguese version of the APAIS will need further validation in a broader surgical population.

## Data Availability

The datasets used and/or analysed during the current study are available from the corresponding author on reasonable request.

## Abbreviations

APAIS: Amsterdam Preoperative Anxiety and Information Scale
HADS: Hospital Anxiety and Depression Scale
STAI: State-trait anxiety inventory
